# Natural Killer Cells Dominate the Hyperacute Lymphocyte Response to Major Trauma and Are Associated with Organ Dysfunction

**DOI:** 10.3390/biom16091228

**Published:** 2026-08-24

**Authors:** Joanna M. Shepherd, Lucy R. Gibb, Hew D. T. Torrance, Joanna Manson, Daniel J. Pennington, Paul Vulliamy, Karim Brohi

**Affiliations:** 1 Centre for Trauma Sciences, Blizard Institute, School of Medicine and Dentistry, Queen Mary University of London, London E1 2AT, UK; 2 Major Trauma Department, St Mary’s Hospital, Imperial College Healthcare NHS Trust, London W2 1NY, UK; 3 Section of Anaesthetics, Pain Medicine & Intensive Care, Department of Surgery & Cancer, Faculty of Medicine, Imperial College London, London W2 1NY, UK; 4 Gloucestershire Hospitals NHS Foundation Trust, Gloucester GL1 3NN, UK; 5 Centre for Immunobiology and Infection, Blizard Institute, School of Medicine and Dentistry, Queen Mary University of London, London E1 2AT, UK; 6 Major Trauma Department, Royal London Hospital, Barts Health NHS Trust, London E1 1FR, UK

**Keywords:** traumatic injury, organ dysfunction, lymphocytes, natural killer cells

## Abstract

The cellular immune response underlying post-injury multiple organ dysfunction syndrome (MODS) remains incompletely described. We hypothesized that early perturbations in innate lymphocyte behavior are critical to the development of MODS in trauma patients. To address this, we examined lymphocyte subsets in a prospective cohort of major trauma patients recruited at a single major trauma hospital. Circulating lymphocytes were profiled with flow cytometry in serial samples drawn within the hyperacute (≤2 h) and acute (24 h, 72 h) post-injury periods. Plasma levels of specific mediators derived from innate lymphocytes were also measured in a larger cohort. We observed a marked hyperacute increase in circulating NK (particularly the CD56^dim^ subset) and Vδ1 cells that were associated with MODS or early mortality. Absolute counts of NK activation markers CD69 and NKG2D were also higher in patients with adverse outcomes, although the proportion of NK cells expressing NKG2D was reduced. Exploratory cluster analyses of NK activating and inhibiting receptors identified patient groups with differing injury characteristics and outcomes. In plasma, patients who developed MODS had significantly higher levels of NK-associated cytotoxic mediators and cytokines. Collectively, these data indicate a specific pattern of hyperacute NK cell activation after major trauma that is characterized by a pattern consistent with cytotoxic lymphocyte activation and is associated with clinical outcome.

## 1. Introduction

Major trauma can result from a range of injury mechanisms including falls, road traffic collisions, and penetrating injuries. The immune system plays a vital role in the recovery of patients after major trauma, but is thought to become dysregulated in patients who develop multiple organ dysfunction syndrome (MODS) [1]. This condition is associated with adverse patient outcomes including infection, mortality, prolonged intensive care unit stays and long-term functional deficits [2,3]. Recent work suggests that the patient trajectory towards MODS or recovery may be determined by initiating immune reactions that occur during the hyperacute period (within 2 h) after trauma [4]. However, individual immune cell responses during this timeframe remain incompletely studied. Detailed examination of immune cell phenotype and behavior during the hyperacute window may facilitate identification of novel therapeutic targets that may reduce the incidence or severity of MODS.

Previous work has identified an association between post-injury lymphopenia or lymphocyte anergy and adverse outcomes such as MODS and mortality [5,6,7]. Mechanisms including lymphocyte exhaustion, apoptosis and migration have been proposed but do not have a consistent scientific evidence base [8,9,10]. The ability to identify the key immune events leading to these observations is limited by delays from injury to sampling, as by 12 h after injury, widespread leucocyte dysregulation has already occurred [11]. Our work has shown that very early differential changes may occur within the circulating lymphocyte compartment that appear to be associated with adverse outcomes [4]. This hyperacute signal is then reversed or lost at later time points (24 h and 72 h after injury), but is thought to be critical for understanding how downstream immune responses subsequently develop [4]. Hyperacute innate lymphocyte activity, particularly of NK cells, appears implicated in the development of MODS [4,7,8,12], however further research is required to directly examine their phenotype and activation status in injured humans. Comprehensive study of different lymphocyte subsets during this period may divulge new insights into potential drivers of maladaptive immune responses following trauma.

We hypothesized that dynamic changes within the circulating lymphocyte compartment occur during the hyperacute window after injury that are associated with MODS. The objectives of this study were to define these changes in the hyperacute and acute periods after injury, and examine their relationship with patient outcomes. Because our previous work identified NK cells as a potentially critical mediator of immune responses [4], we further examined changes in select NK activating (NKG2D, CD69) and inhibitory (NKG2A, KIR2DL3) receptor profiles, as well as circulating levels of NK-associated mediators in plasma.

## 2. Materials and Methods

### 2.1. Study Setting and Patient Selection

This investigation was performed as part of the prospective cohort study Activation of Coagulation and Inflammation in Trauma (ACIT2) [4,7]. All adult patients (aged over 15 years) at a single major trauma center who met criteria for trauma team activation were eligible for enrollment into ACIT2. Exclusion criteria were presentation more than 2 h from injury, interhospital transfer, or administration of more than 2000 mL crystalloid prior to arrival. Approvals for ACIT2 were granted by the National Health Service (NHS) Research Ethics Committee (REC) (reference 07/Q0603/29). All patients or their representatives provided written informed consent for study inclusion.

For flow cytometry studies, in order to focus resources on the most severely injured patients, additional criteria for enrollment included: activation of the major hemorrhage protocol; requirement for surgical or radiological intervention; requirement for critical care admission; or if the attending research fellow judged the sustained injuries to be severe (defined as an injury severity score (ISS) greater than or equal to 9, in line with existing literature [12,13]). Patients with isolated traumatic brain injury (TBI) and previous use of immune modulating medications including steroids were excluded. Patients were retrospectively excluded if they had an ISS less than 9. Healthy volunteers were recruited as a comparator group (NHS REC: 13/LO/0363).

### 2.2. Data Collection and Definitions

Data on patient demographics, baseline admission and injury characteristics, treatment and outcomes were prospectively collected on a daily basis. Injury severity score was used as a measure of injury load and base deficit (BD) was used as a measure of systemic hypoperfusion. Peripheral blood samples were obtained at admission (within 2 h after injury), and at 24 ± 2 h and 72 ± 12 h after injury. Patient outcomes including MODS, mortality (24 h and 30-day), time to recovery (TTR), incidence of infection, ventilator-free days, vasopressor-free days, and critical care and hospital length of stay were prospectively recorded. MODS was defined as the presence of a Sequential Organ Failure Assessment (SOFA) score of greater than or equal to six [2,14]. TTR was defined as the time taken (days) for the SOFA score to fall below the MODS threshold of six [2].

### 2.3. Outcome Groups

The primary clinical comparator groups were MODS vs. No MODS amongst patients surviving more than 24 h. Early deaths under 24 h served as a secondary comparator, as MODS is not evaluable in this cohort.

### 2.4. Flow Cytometry

Peripheral blood mononuclear cells (PBMCs) were isolated from CPT^TM^ tubes (Becton Dickinson, Wokingham, UK) using the manufacturer’s instructions within 2 h of collection. Freshly prepared mononuclear cells were washed in phosphate buffered saline (Thermofisher Scientific, London, UK) twice and re-suspended in staining buffer (Dulbecco’s phosphate buffered saline, with 2% Fetal Bovine Serum, (Stemcell, Cambridge, UK)) prior to staining. Lymphocyte sub-populations were identified using combinations of the following antibodies in four separate panels (Supplemental Table S1). Details of antibodies used for flow cytometry analysis, including fluorochrome, clone, company and catalog number are provided in Supplemental Table S2. NK cells were classified as NK^dim^ or NKbright based on surface expression of CD56. NK surface activation and inhibition receptors were quantified using the following antibodies: CD69, NKG2D, KIR2DL3/CD158b, NKG2A/CD159a (Supplementary Table S2). Isotype controls were used to facilitate gating for CD69-APC and NKG2D-PE-Cy7. After staining, samples were washed in PBS and fixed in 1% paraformaldehyde solution for 25 min. Acquisition was performed using an LSR II flow cytometer (Becton Dickinson, UK). Healthy volunteer samples were collected and processed using the same protocol as trauma patients.

### 2.5. Plasma Proteomics

Blood was collected into P100 vacutainers (Becton Dickinson, Wokingham, UK) and centrifuged at 2500× *g* for 10 min. The plasma fraction was then isolated and stored at −80 °C prior to analysis. Plasma proteins were measured using the SomaScan platform (SomaLogic, Inc., Boulder, CO, USA) as previously described [14] and are reported as relative fluorescence units (RFU).

### 2.6. Data Analysis

Flow cytometry data were analyzed using FlowJo (Versions 10 and 11 Tree Star Inc., Ashland, OR, USA). The gating strategy is illustrated in Supplemental Figure S1. The results were subsequently exported into a Microsoft Excel (Microsoft, Inc., Redmond, WA, USA) master spreadsheet and analyzed using Prism (Versions 7.0 and 11, GraphPad, Boston, MA, USA). Demographic and outcome data were analyzed using IBM SPSS Statistics for Macintosh (Version 25). Absolute counts for lymphocyte subsets were obtained by multiplying the percentage of the total lymphocyte population acquired by flow cytometry by the absolute lymphocyte count obtained from routine hospital laboratory tests. Data were tested for normality using the Shapiro–Wilk test. Summary data are principally reported as median (interquartile range) and analyzed using non-parametric statistics unless otherwise stated.

To further explore patterns of NK activation and inhibition profiles at the ≤2 h timepoint, patients who had complete data sets for NKG2D, CD69, NKG2A and KIR2DL3 receptors were entered into an exploratory unsupervised hierarchical cluster analyses using MatLab (Version R2017b, MathWorks, Inc., Natick, MA, USA). Data for each receptor were normalized prior to clustering so that the clustering primarily reflected underlying biological variability rather than variability within the individual assays. Normalization was performed using the following equation: (x − m)/(x_max_ − x_min_) where x is the observed value to be standardized, m is the reference (healthy volunteer) population mean, and x_max_ and x_min_ are the maximum and minimum observed values for the sample dataset. This range-based approach, rather than standardization by standard deviation (e.g., z-score), was used because z-score normalization can sometimes reduce genuine variability within the dataset [15]. The clustering algorithm utilized Euclidean distances as the similarity measure and Ward linkage as the clustering method. The number of clusters was selected by visual interpretation of the dendrograms. This methodology was based on previous work within our unit that explored clustering techniques within comparable trauma datasets [16,17].

## 3. Results

### 3.1. Patient Characteristics

A total of 65 trauma patients and 18 healthy volunteers were included in the flow cytometry cohort (Supplemental Figure S2). The majority of trauma patients were male (75%) who sustained blunt trauma (83%) and activated the major hemorrhage protocol (80%; Table 1). The median age was 42 years and the median ISS was 25. The median age for the healthy volunteers was 32 (IQR 29 to 37) and 61% were male. Patients with MODS (vs. No MODS) were more severely injured (ISS 33 vs. 17, *p* = 0.001) and more likely to have sustained traumatic brain injury (34% vs. 0%, *p* = 0.005). They were also more shocked (BD 7.4 mmol/L vs. 1.1 mmol/L, *p* < 0.001), had worse admission GCS scores (13 vs. 15, *p* = 0.001), and had higher crystalloid and transfusion requirements (24 h crystalloid 3800 vs. 143 mL; 24 h red blood cells 5 vs. 1 units; *p* < 0.001).

### 3.2. Dynamic Changes in Circulating Lymphocyte Subsets During the Hyperacute and Acute Phases After Injury

Total lymphocyte counts were not significantly different to healthy controls at admission (within 2 h), but progressively declined below the lower limit of normal at 24 and 72 h after injury (Table 2). When individual lymphocyte subsets were analyzed we found that NK cell counts more than doubled at the hyperacute timepoint relative to controls (4.6 vs. 2.1 × 10^8^/L, *p* < 0.001), and in some cases accounted for over 50% of the circulating lymphocyte compartment. However, by 24 h and 72 h after injury, they were significantly reduced from circulation, reducing their overall numbers by 8-fold from admission, and representing only a quarter of the number that would be expected during health (0.58 × 10^8^/L at 72 h, *p* < 0.001 vs. 2 h). The initial rise in total NK cell counts was driven primarily by increases in the cytotoxic NK^dim^ subset, whose absolute counts were markedly elevated at ≤2 h compared to controls (3.5 vs. 2.0 × 10^8^/L, *p* = 0.004). NK^bright^ cells trended towards higher counts at admission but the difference was not statistically significant (0.14 vs. 0.2 × 10^8^/L *p* = 0.068). Both subsets had declined by 24 h, though the change was greatest in the NK^dim^ subset, whose numbers had reduced 5-fold, relative to a 2-fold reduction in NK^bright^ cell counts.

None of the other subsets demonstrated an overall increase during the hyperacute period compared with controls, but CD4+ T cell counts were significantly reduced within 2 h of injury (6.7 vs. 11 × 10^8^/L, *p* = 0.002). All T cell subsets declined by 24–72 h (Table 2), reflecting the overall lymphopenia, whereas B cell counts were not significantly different from controls at any time point. Overall, this data shows hyperacute, selective mobilization of circulating NK cells after injury followed by a generalized and progressive lymphopenia over the subsequent 72 h.

### 3.3. Hyperacute Changes in Circulating Lymphocyte Subset Counts Are Associated with MODS and Mortality

**Figure 1:**
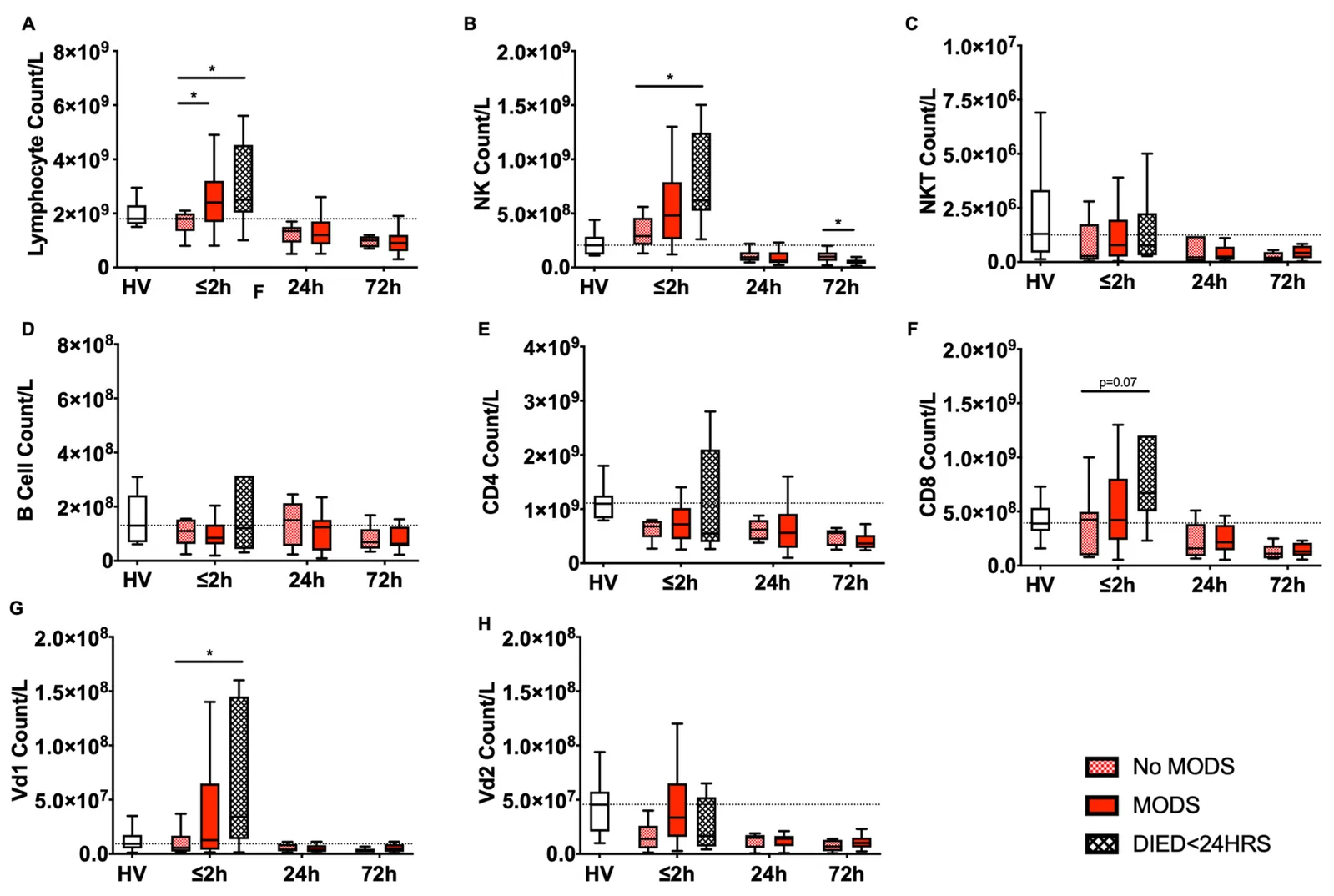
Relationship between lymphocyte subset counts and development of Multiple organ dysfunction syndrome (MODS). Total lymphocyte and individual subsets were quantified by flow cytometry in healthy volunteers (HV, total *n* = 18) and injured patients who did not develop MODS (total *n* = 18), patients who developed MODS (total *n* = 35) and patients who died within 24 h (total *n* = 12). (**A**): Total lymphocyte count (HV, *n* = 18; ≤2 h, *n* = 63; 24 h, *n* = 47; 72 h, *n* = 47). (**B**): Natural Killer (NK) cell count (HV, *n* = 18; ≤2 h, *n* = 63; 24 h, *n* = 39; 72 h, *n* = 40). (**C**): NKT cell count (HV, *n* = 18; ≤2 h, *n* = 43; 24 h, *n* = 25; 72 h, *n* = 24). (**D**): B cell count (HV, *n* = 14; ≤2 h, *n* = 30; 24 h, *n* = 17; 72 h, *n* = 16). (**E**): CD4 T-cell count (HV, *n* = 13; ≤2 h, *n* = 31; 24 h, *n* = 18; 72 h, *n* = 16). (**F**): CD8 T-cell count (HV, *n* = 13; ≤2 h, *n* = 31; 24 h, *n* = 17; 72 h, *n* = 16). (**G**): Vδ (Gamma-delta)-2 count (HV, *n* = 18; ≤2 h, *n* = 46; 24 h, *n* = 26; 72 h, *n* = 24). (**H**): Vδ1 count (HV, *n* = 18; ≤2 h, *n* = 46; 24 h, *n* = 26; 72 h, *n* = 24). * *p* < 0.05; ≤2 h Kruskal–Wallis test with Dunn’s post hoc corrections comparing No MODS vs. MODS vs. early deaths; 24 h and 72 h Mann–Whitney U-test comparing No MODS vs. MODS. Dashed lines indicate median value from healthy volunteers.

We next examined the relationship between lymphocyte subset dynamics and clinical outcomes. In the hyperacute window, total lymphocyte counts were elevated in patients who developed MODS or died within 24 h compared to those who did not develop MODS (Figure 1A). This pattern was primarily attributable to a rise in NK cell counts (No MODS 2.9 [2.1–4.6]; MODS 4.8 [2.6–7.9]; died <24 h 6.2 [5.2–12.5] × 10^8^/L, *p* = 0.019; Figure 1B and Supplemental Figure S3). When NK^dim^ and NK^bright^ subsets were analyzed separately, we found this association was dominated by the NK^dim^ subset (Supplemental Figure S4A). Conversely, there were no statistically significant differences in B cells, CD8+ T cells, CD4+ T cells, Vδ2 cells, or NKT cell counts across the different patient groups (Figure 1C–G). Vδ1 cells were higher in patients who developed adverse outcomes (No MODS 0.06 [0.02–0.18]; MODS 0.13 [0.04–0.65]; died <24 h 0.34 [0.14–1.5] × 10^8^/L, *p* = 0.035; Figure 1H), although as expected, their circulating concentrations were an order of magnitude lower than NK cells. At 72 h, patients with MODS had significantly lower NK counts (MODS 0.5 [0.3–0.7] × 10^8^/L vs. No MODS 1.0 [0.6–1.4], *p* = 0.021). There were no significant changes in counts between the groups for any of the other lymphocyte subsets at 24 or 72 h.

### 3.4. NK Activation Markers Show Distinct Temporal Signatures After Major Trauma

**Figure 2:**
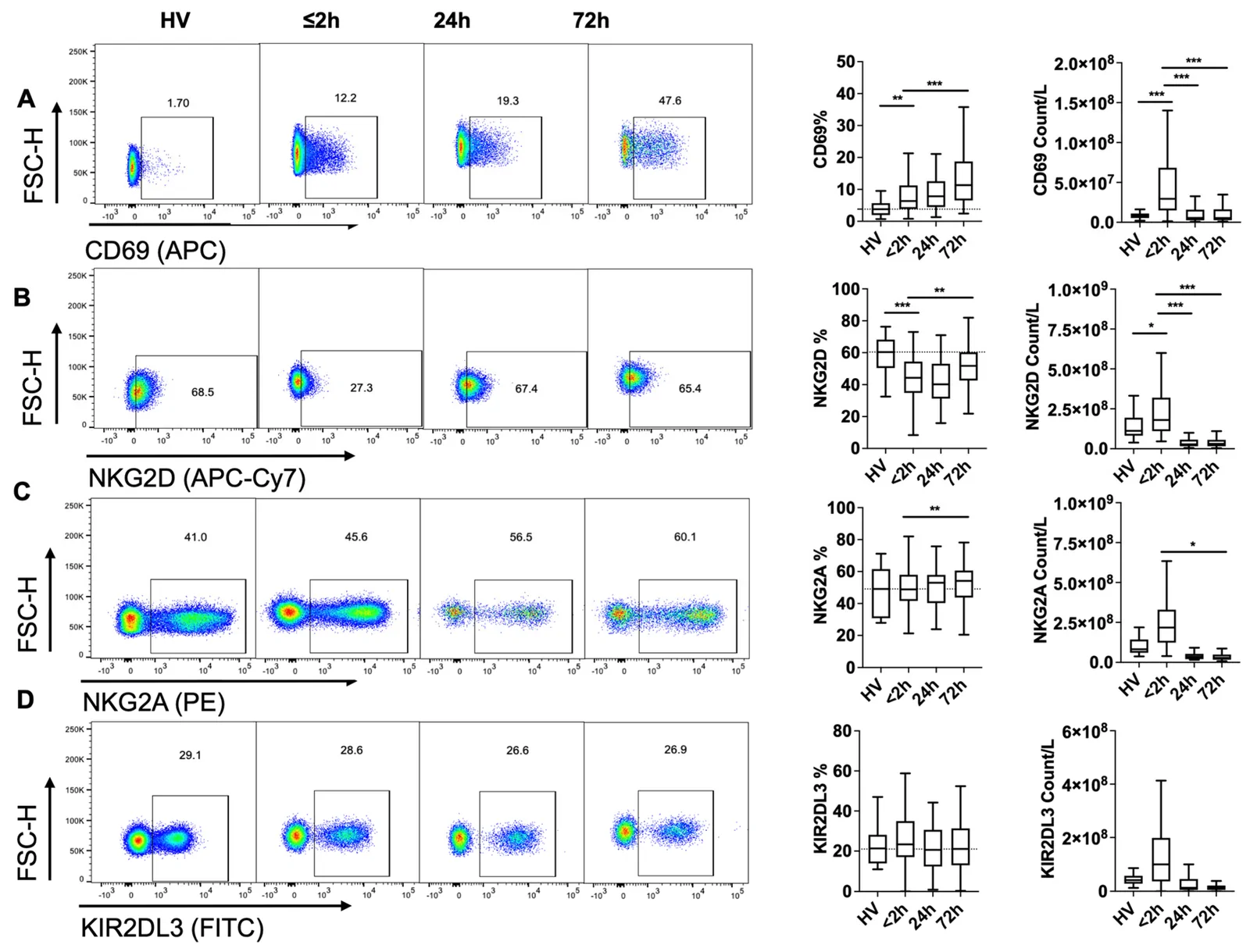
Dynamic changes in surface expression of NK cell activation and inhibition receptors in the hyperacute and acute phases of injury. Representative flow cytometry plots (left panels) show (**A**) a patient whose CD69 increased over time (ISS 34, BD 4); (**B**) a patient with an initial reduction in NKG2D followed by recovery towards normal (ISS16, BD18); (**C**) a patient who had a small increase in NKG2A over time (ISS 59, BD 9.6); and (**D**) a patient in whom KIR2DL3 did not change (ISS 16, BD 18). Box plots (right panels) show median with interquartile range and 10th to90th percentiles in healthy volunteers (HV) and patients at ≤2 h, 24 h and 72 h after injury. * *p* < 0.05, ** *p* < 0.01, *** *p* < 0.001, Kruskal–Wallis test with Dunn’s post-test correction. Dashed lines indicate median value from healthy volunteers.

Given the dramatic hyperacute elevations in NK cells, we then examined the number and proportion of NK cell cells that were positive for surface activating and inhibiting receptors. We found higher numbers of circulating NK cells that were positive for activating receptors CD69 (2.9 [1.5–6.8] vs. 0.8 [0.5–1.1] × 10^8^/L, *p* < 0.001) and NKG2D (1.8 [1.1–3.2] vs. 1.1 [0.8–1.9] × 10^8^/L, *p* < 0.001) at admission relative to controls, broadly reflecting the changes seen in the total NK cell population. Increases in admission counts of NK cells that were positive for inhibitory receptors NKG2A and KIR2DL3 were not statistically different to controls. When the receptors were analyzed as proportions of the NK population, we found the proportion of NK cells that were positive for the early activation maker CD69 was elevated at admission (6.4 vs. 3.8%, *p* = 0.008) and rose progressively at 24 and 72 h (Figure 2A). Conversely, the proportion of NK cells that were positive for NKG2D was decreased at admission (44.3 vs. 60.4%, *p* < 0.001) and remained low at later timepoints (Figure 2B). The proportion of NKs expressing inhibitory receptors NKG2A and KIR2DL3 was not significantly different at any timepoint (Figure 2C,D). Collectively, these data demonstrate hyperacute mobilization of high numbers of NK cells expressing activating receptors, with a potential paradoxical reduction in the proportion of NK cells that were positive for NKG2D, and a subsequent loss of NK cells from circulation.

### 3.5. Associations Between NK Surface Receptor Profiles and Clinical Outcomes

High numbers of NKG2D+ NK cells during the hyperacute period were associated with adverse outcomes (Supplemental Figure S4B) including both MODS and mortality (No MODS 11, MODS 21, Died <24 h 36 × 10^7^; *p* < 0.001). This reversed by 72 h where lower numbers of NKG2D+ NK cells were associated with MODS (No MODS 5.6, MODS 2.9 × 10^7^; *p* = 0.020). Similar findings were identified for CD69+ cells at the ≤2 h time point (No MODS 1.6, MODS 3.0, Died <24 h 9.8 × 10^7^; *p* = 0.001). No associations between adverse outcome and the number of NK cells expressing inhibitory receptors NKG2A and KIR2DL3 were identified. When proportions of NK cell receptors were examined, we found lower proportions of NKG2A+ NK cells at ≤2 h appeared to be associated with worse outcomes (No MODS 56%, MODS 48%, Died <24 h 43%: *p* = 0.026), but no other associations were identified.

### 3.6. NK Receptor Profiles Identify Clusters of Patients with Different Outcomes and Clinical Characteristics

**Figure 3:**
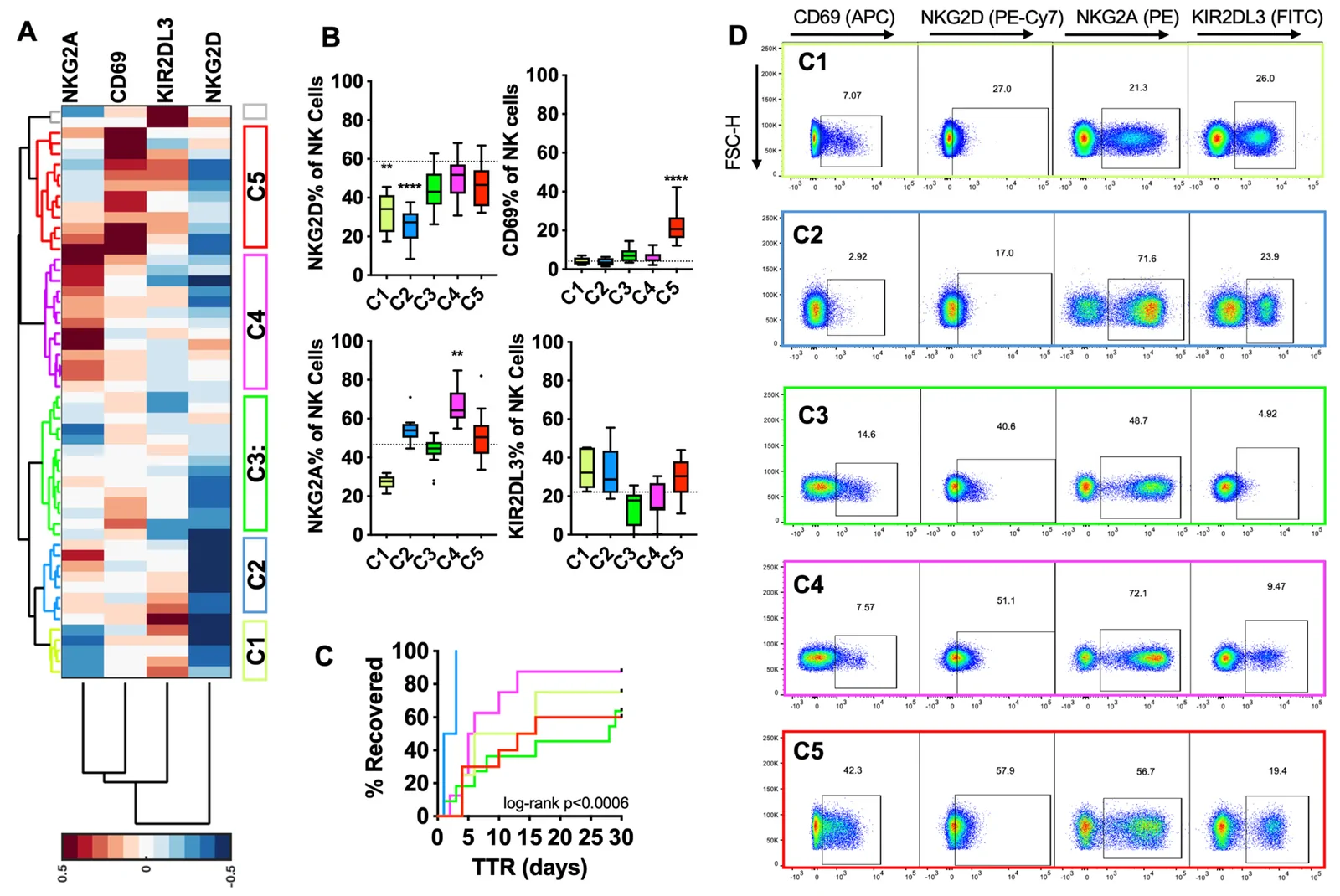
NK cell surface activation and inhibition receptors show five distinct clusters in the hyperacute post-injury window. NK cell surface receptor positivity was quantified by flow cytometry in injured patients with complete data on four markers (NKG2A, CD69, KIR2DL3, NKG2D; *n* = 54), normalized against healthy volunteer values (*n* = 18), and subjected to unsupervised hierarchical clustering. (**A**): Dendrogram and heatmap of hierarchical clustering output. (**B**): Proportion of NK cells expressing each receptor within clusters C1–C5; dotted line indicates healthy volunteer median. (**C**): Kaplan–Meier time-to-recovery curves for C1–C5; comparisons by Mantel–Cox log-rank test. (**D**): Representative flow cytometry plots for each cluster: C1 (BD 26, ISS unknown, died <24 h); C2 (ISS 16, BD 0.6, TTR 0); C3 (ISS 12, BD 22, TTR 29); C4 (ISS 9, BD 5.6, TTR 0); C5 (ISS 38, BD 7.6, TTR 4). ** *p* < 0.01, **** *p* < 0.0001 vs. healthy volunteers; Kruskal–Wallis test with Dunn’s post hoc correction.

To discover whether combinations of different receptor proportions during the hyperacute period could identify patients who developed different outcomes, we performed an exploratory hypothesis generating cluster analysis using patients who had a complete admission data set for activation and inhibition receptors (*n* = 54). This generated 5 main clusters (Figure 3): C1, characterized by low proportions of NKG2D+ and NKG2A+ NK cells relative to controls (NKG2D+ 34 vs. 61%, *p* = 0.002; NKG2A+ 28 vs. 50%, *p* = 0.064); C2, characterized by very low proportions of NKG2D+ NK cells (27 vs. 61%, *p* < 0.001); C3, which did not differ substantially from controls, except for a trend towards higher proportions of CD69+ NK cells (6.9 vs. 3.5%, *p* = 0.066); C4, characterized by high proportions of NKG2A+ NK cells (64 vs. 50%, *p* = 0.002); and C5, characterized by very high proportions of CD69+ NK cells (21 vs. 3.5%, *p* < 0.0001). The five clusters also differentiated injury profiles and outcomes (Supplemental Table S3), with C2 and C4 exhibiting the shortest recoveries, and C1, C3 and C5 exhibiting the poorest recoveries (Figure 3C). Statistical comparisons demonstrated that the greatest differences were between C2 and C5 clusters, where C5 patients were more severely injured and shocked, and had higher transfusion requirements; they were more likely to develop MODS and infection, have greater critical care requirements. Similar differences were noted between C4 and C5 clusters, and between C2 and C3 clusters. This illustrates that NK receptor profiles on admission are potentially able to differentiate clusters of patients with different outcomes and clinical characteristics.

### 3.7. Circulating Levels of NK-Associated Mediators Are Increased in Patients with Adverse Clinical Outcomes

**Figure 4:**
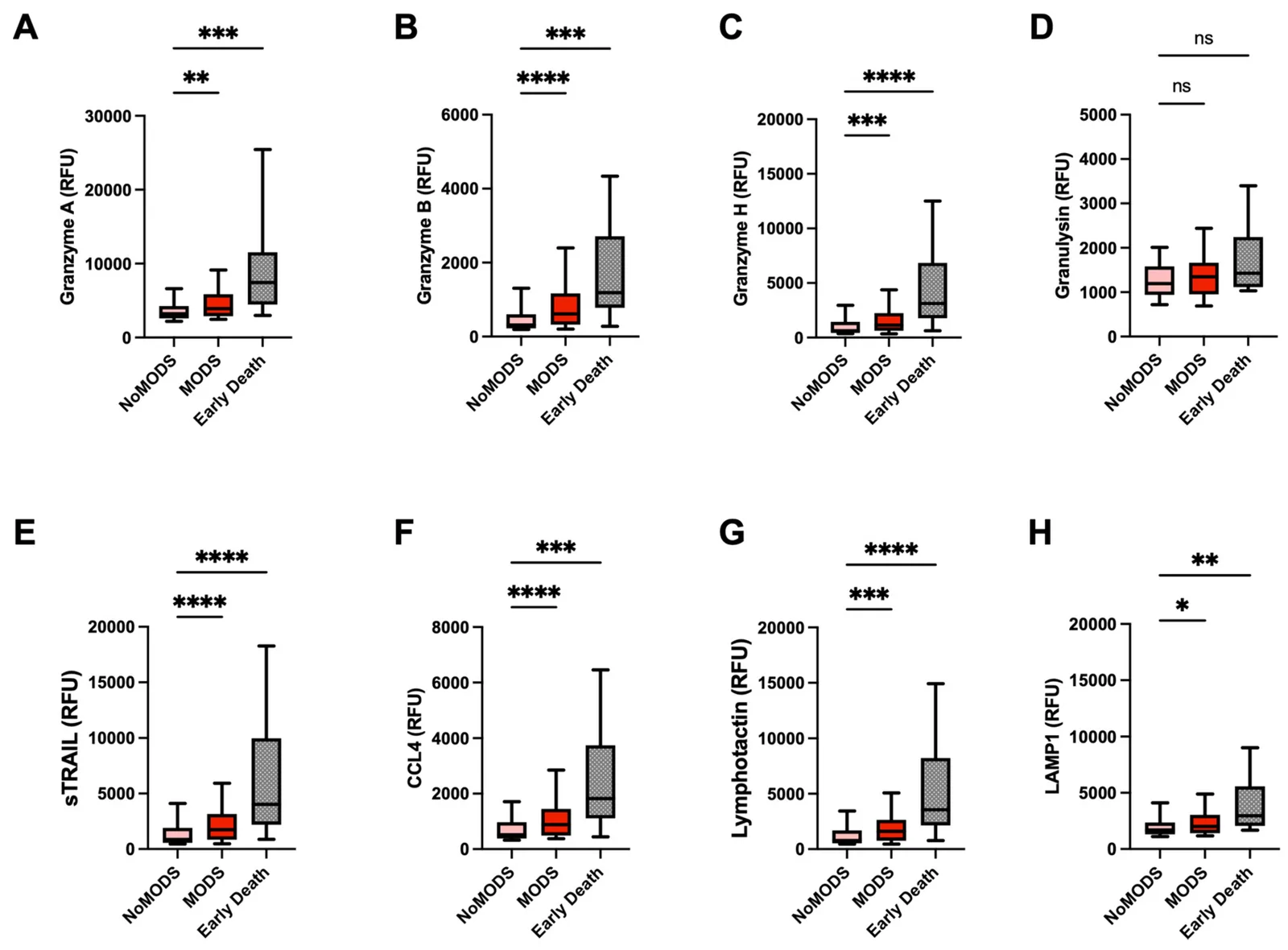
Plasma levels of NK-associated mediators are elevated in patients with adverse outcomes at the hyperacute timepoint. Plasma proteins were measured in platelet-poor plasma obtained within 2 h of injury using the SomaScan platform (SomaLogic, Inc.). Patients are grouped as No MODS (*n* = 317), MODS (*n* = 83), and early death within 24 h (*n* = 12). Cytotoxic granule mediators: (**A**) Granzyme A; (**B**) Granzyme B; (**C**) Granzyme H; (**D**) Granulysin. NK-associated cytokines and degranulation marker: (**E**) sTRAIL; (**F**) CCL4; (**G**) Lymphotactin; (**H**) LAMP1. Box plots show median, interquartile range and 10th to 90th percentiles. * *p* < 0.05, ** *p* < 0.01, *** *p* < 0.001, **** *p* < 0.0001 vs. No MODS; ns, not significant (*p* > 0.05). Kruskal–Wallis test with Dunn’s multiple comparisons test. RFU, relative fluorescence units.

Finally, we examined associations between NK cell-derived mediators in plasma at the hyperacute timepoint and clinical outcome. These studies were collected in a separate cohort of trauma patients recruited to ACIT2 (*n* = 412), of whom 108 developed MODS and 12 died within 24 h; detailed characteristics are shown in Supplemental Table S4). Patients who developed MODS or who died within 24 h had significantly higher plasma levels of the NK-associated cytotoxic mediators Granzyme A, Granzyme B and Granzyme H (Figure 4A–C), although there was no significant difference in granulysin levels (Figure 4D). Similarly, circulating levels of NK-associated cytokines sTRAIL, lymphotactin, CCL4 (Figure 4E–G) and the lysosomal maker LAMP-1 (Figure 4H) were higher in patients with adverse outcomes. Collectively this data indicates that the hyperacute elevations in NK cells are linked to increased NK cell degranulation and cytotoxic mediator release in patients with adverse outcomes.

## 4. Discussion

In this study of critically injured trauma patients, dynamic changes in circulating lymphocyte populations were evident over the first 72 h of injury, commencing within the hyperacute timeframe. Cytotoxic innate lymphocytes including NK cells (particularly CD56^dim^ NK cells) and Vδ1 cells were differentially elevated in the hyperacute window and were associated with adverse outcomes. These changes correlated with plasma signatures of NK cell derived mediators. To our knowledge, this is the first study that specifically profiles the expression of NK activating and inhibiting cell surface receptors in a human trauma cohort using flow cytometry. Exploratory analyses suggested that changes in the absolute counts or proportions of these receptors during the hyperacute period could potentially discriminate patients with adverse outcomes. Collectively this work highlights a potential role for cytotoxic innate lymphocytes in development of organ injury and adverse outcome after major trauma.

Associations between early circulating NK cell rises and poor outcomes following trauma (including MODS and mortality) are described in previous studies [7,18], and supported by whole blood leucocyte gene expression studies in trauma patients [4]. Similar associations are also described in other critically ill patient cohorts including sepsis [19,20]. Our study supports and develops these findings, as well as illustrating comparable patterns in other cytotoxic innate lymphocytes including Vδ1+ cells. We further demonstrate that NK cells (in particular the cytotoxic NK^dim^ subset) undergo a dynamic shift from supranormal levels at ≤2 h from injury, to subnormal levels by 24 h, consistent with previous findings [12,21], and this was also associated with adverse outcome. Previous work has comprehensively examined the hyperacute period in conjunction with later time points in a human trauma cohort [12]. This showed that all lymphocyte groups (including NK, T and B cells) were elevated in the hyperacute period, and subsequently declined. Further work is required to determine whether innate lymphocyte elevations within the hyperacute period are differential (as in this work) or consistent with the global behavior of all lymphocytes [12,22].

The mechanisms underlying differential changes in lymphocyte subsets during the hyperacute period after trauma are unclear. Small studies show that co-culture of PBMCs with DAMPs may drive a selective NK subset increase [22]. However, selective increases in NK populations have also been observed under other conditions of acute stress. Acute exercise induces a rapid increase in circulating NK cells, particularly mature cytotoxic cell subsets [23,24,25,26]. Circulating NK cells have also been shown to be significantly elevated within minutes of acute psychological stress, in the form of marital conflict [27]. This pattern appears to be, at least in part, mediated by catecholamine signaling, as β2-adrenergic receptor blockade blunts exercise induced NK cell mobilization [24], while exogenous catecholamine administration results in selective mobilization of NK^dim^ and Vδ lymphocytes into the periphery within minutes of administration [28]. In the context of trauma, the profound catecholamine surge associated with the systemic stress response [29] may therefore mobilize lymphocytes, predominantly mature cytotoxic NK cells, from reservoirs such as bone marrow, spleen and lungs into the peripheral circulation [30]. Whether these mechanisms underlie the early lymphocyte subset changes observed in human trauma are the hypotheses for further research.

The differential behavior of Vδ1 and Vδ2 cells also warrants further consideration. Vδ1 cells are predominantly tissue resident intra-epithelial cells that predominantly recognize self-induced stress ligands including MICA/MICB and ULBPs NKG2D. This T-cell receptor (TCR) independent pathway is shared with NK cells and therefore may provide a mechanistic link [31]. Whereas Vδ2 cells (which represent the bulk of circulating Vδ T cells) are predominantly TCR dependent and recognize microbial and self-phosphoantigens. The mechanisms underlying the subsequent decline in NK and Vδ1 numbers also lack a consistent evidence base, but could include re-margination once catecholamine levels decline, migration to areas of soft tissue injury, or activation induced apoptosis. Further studies could provide clarity through further tissue sampling and apoptosis assays [8].

When NK activating and inhibitory receptors were examined, we found adverse outcomes were associated with hyperacute increases in activating receptor counts (i.e., CD69, NKG2D) and not inhibitory receptors (NKG2A and KIR2DL3). Exploratory cluster analysis on the proportions of NK receptors broadly supported these results. The potential association between adverse outcome and NK activation in trauma patients is consistent with transcriptomic data reported previously [4], and is also reflected in sepsis studies [32,33]. However, depressed NK function is also associated with impaired pathogen clearance, and has also been associated with MODS in small human trauma cohort studies [34,35]. Whether NK activation is beneficial or detrimental may depend on multiple factors, including timing in relation to the initial insult and the type of pathogen encountered [33]. Absolute numbers of NK cells that were NKG2D+ were increased in this study, however there was a global reduction in the proportion of NK cells that were NKG2D+. NKG2D ligands, including major histocompatibility complex class I related chains A and B (MIC-A and MIC-B) and UL-16 binding proteins, are classically expressed on stressed or transformed cells. It is possible that under conditions of acute stress, NKG2D downregulation prevents inappropriate NK cytotoxicity against bystander healthy cells [36,37]. Trauma patients who retain higher numbers or proportions of NK cells that are NKG2D+ may therefore be at risk of increased organ injury as a result of an initially over-exuberant NK response. The observation that some patients appeared to have lower proportions of NK cells that were NKG2D+ in this trauma cohort warrants further study, both to validate the finding, and to explore the mechanism of potential downregulation.

The cluster analysis in this study identified a cluster of patients defined by very high proportion of NK cells that were positive for the early activation receptor CD69 during the hyperacute period. These patients were amongst the most shocked patients in the cohort and suffered poor outcomes. The ligands for CD69 are not fully defined [38,39] therefore it is unclear if its expression following trauma directly influences NK behavior or simply represents non-specific NK activation. Previous studies show that stimulation of NK cells with anti-CD69 monoclonal antibodies independently trigger NK cytotoxicity against target cells, and that addition of anti-CD94/NKG2A blocks this effect [38]. Trauma patients with high numbers of unopposed CD69+ cells may therefore be at higher risk of cytotoxic NK mediated tissue destruction. We also observed that the proportion (but not absolute count) of circulating CD69+ NK cells increased over the subsequent 24–72 h. This may represent the sustained activation of NK cells remaining in circulation, NK cell anergy post-stimulation, or selective depletion of CD69 negative cells from circulation [40].

When inhibitory receptors were examined, patients with higher proportions of NKG2A+ NK cells within 2 h of injury appeared to have lower rates of MODS and early death. One potential hypothesis is that NKG2A plays a role in tempering potentially destructive NK activity. The key ligand for NKG2A is HLA-E, expressed on the majority of healthy cells. Tissue-specific upregulation of HLA-E in response to stimulation may occur as an immunoprotective mechanism against unwanted injury by NK cells. Endothelial stimulation with pro-inflammatory cytokines has been shown to upregulate both membrane-bound and soluble HLA-E, and that NKG2A blockade promoted NK mediated lysis of activated endothelial cells [41]. To investigate this as a potential mechanism in trauma, further study could commence by examining soluble HLA-E in trauma patients, for instance using previously described ELISA methodology [42].

This study did not show any associations between NK surface expression of KIR2DL3 and patient outcome. KIR receptors are key regulators of NK immune tolerance, however they are highly polymorphic, with polymorphism affecting their expression, affinity and function [43]. It is possible that by restricting our investigation to the surface expression of one KIR member provides insufficient detail to identify interactions that may lead to poor outcome.

Analysis of plasma mediators supports the observation that NK cells appear to shift towards a cytotoxic phenotype in the hyperacute window, with high levels of granzymes A, B and H in patients who developed MODS or who died early after injury. These mediators are released from NK cells upon stimulation and are consistent with our flow cytometry data indicating higher relative and absolute numbers of activated NK cells [44]. Our observation that granulysin levels do not follow the same pattern as the granzymes may be explained by the fact that its release is more tightly regulated than other granule components [45], whereas granzymes can be released following immune synapse-independent cytokine stimulation [44]. In addition, we found elevations in the NK derived cytokines sTRAIL, lymphotactin and CCL4, suggesting that NK cells are not only cytotoxic, but also capable of shaping downstream inflammation through promoting cell death, recruiting other immune cells including dendritic and myeloid cells, and supporting a transition to a sustained inflammatory response [46,47,48].

There are several limitations of this study. The cohort of patients is relatively small, although is comparable to larger flow cytometry studies conducted in trauma patients to date, and has the advantage of sampling both in the hyperacute time frame and across the subsequent 72 h at defined time points. Healthy volunteers were not specifically matched to patients, which could affect comparisons. Loss of follow-up samples (for instance, due to death, discharge or patients declining further blood draws) may have resulted in participant bias for comparisons with later time points. Owing to the logistical challenges of prospective studies in injured patients, the cohort with plasma proteomic measurements was separate to the flow cytometry cohort. Clinical characteristics including injury severity were different (although notably the population with MODS had similar characteristics in both cohorts), and thus this data should be viewed as supportive rather than directly indicating that the NK cells measured by flow are producing these mediators. We could not exclude patients who had pre-sample blood transfusions, due to protocols recommending blood product delivery in the pre-hospital setting to patients with suspected major hemorrhage. It is possible that absolute lymphocyte counts may have been influenced by pre-sampling interventions. Owing to the small sample size, we did not perform multivariable modeling to elucidate whether NK cell phenotype is associated with MODS independently of injury severity, shock or head injury. Therefore it is difficult to fully isolate whether associations with adverse outcome are true associations or simply reflections of worse injuries; the results are also dependent on the definitions of MODS used in this study. The dataset used for the cluster analysis is comparatively small, and the analysis is exploratory. This should be viewed as hypothesis-generating, and the clusters generated require further validation in a larger cohort with formal stability testing and potentially integration with other datasets [49,50]. Larger sample sizes would also permit use of data mining approaches that may unravel non-linear and interactive relationships between outcomes and cell observations. Nevertheless, the data presented provide a platform for further hypothesis testing in larger cohorts of patients. Further preclinical research is required to investigate the potential causative role of cytotoxic NK cell expansion in post injury organ failure, and identify specific biomarkers of NK activation that could rapidly identify the risk of MODS. This could unlock precision clinical trials using emerging treatments, such as dasatinib, to pharmacologically modulate NK cell activation and potentially protect against post-injury organ dysfunction.

## 5. Conclusions

In conclusion, this study provides detailed insights into the dynamic and differential changes in circulating lymphocytes that occur after injury. Our results highlight that changes in NK cells predominate in the hyperacute window, and that these changes are associated with adverse clinical outcomes. The extent to which these processes reflect an ‘appropriate’ evolved response to enhance immune surveillance that becomes maladaptive in the context of severe injuries remains an open question. Further research is required to determine the mechanisms underlying this rapid NK mobilization and distinct activation signature, as well as the potential causative role of cytotoxic NK cell expansion in post injury organ failure.

## Figures and Tables

**Table 1:** Cohort demographics and outcomes.

	All Trauma	No MODS	MODS	Died < 24 h	*p*-Value
**N**	65	18	35	12	
**Demographics**					
Age	42 (29–55)	38 (24–51)	42 (29–57)	45 (28–61)	0.252
Male (*n*)	75% (49)	83% (15)	69% (24)	83% (10)	0.248
**Injury Characteristics**					
Blunt (*n*)	83% (54)	67% (12)	89% (31)	92% (11)	0.054
ISS	25 (16–38)	17 (9–25)	33 (20–43)	34 (16–45)	**0.001**
% TBI	20% (13)	0	34% (12)	9% (1)	**0.005**
**Baseline Physiology**					
HR (beats/min)	109 (80–125)	91 (77–109)	114 (90–128)	107 (73–120)	**0.020**
SBP (mmHg)	115 (79–131)	126 (117–146)	112 (82–129)	47 (0–95)	**0.040**
GCS	14 (6–15)	15 (14–15)	13 (7–15)	3 (3–6)	**0.001**
pH	7.24 (7.12–7.37)	7.38 (7.34–7.39)	7.23 (7.16–7.28)	6.88 (6.74–6.97)	**<0.001**
BD	5.7 (2.3–13.2)	1.1 (0.1–4.3)	7.4 (3.7–10.0)	18.1 (15.1–28.2)	**<0.001**
**Interventions**					
MHP (*n*)	80% (52)	56% (10)	86% (30)	100% (12)	**0.016**
TXA (*n*)	80% (52)	50% (9)	91% (32)	92% (11)	**0.001**
Pre-baseline RBC	1 (0–3)	0 (0–0)	1 (0–3)	4 (1–5)	**0.002**
Pre-baseline CSL	0 (0–500)	0 (0–50)	100 (0–500)	400 (0–775)	**0.034**
24 h RBC	4 (1–8)	1 (0–2)	5 (2–8)	17 (7–33)	**<0.001**
24 h CSL	2520 (1000–4143)	143 (0–3250)	3800 (2253–4900)	132 (0–2125)	**<0.001**
**Outcomes**					
28 d Mortality (*n*)	23% (15)	0	8.8% (3)	100% (12)	0.194
24 h Mortality (*n*)	18% (12)	0	0	100% (12)	-
TTR (days)	5 (0–29)	0 (0–0)	6 (3–16)	-	**<0.001**
Infection (*n*) ^a,b^	42% (22)	0	65% (22)	-	**<0.001**
Ventilator FD	26 (0–30)	30 (30–30)	24 (14–28)	-	**<0.001**
Critical care FD	20 (0–27)	30 (27–30)	16 (2–24)	-	**<0.001**
Hospital FD	0 (0–17)	20 (14–25)	0 (0–9)	-	**<0.001**

Data presented as median (interquartile range) unless specified. *p*-values correspond to comparisons of MODS vs. No MODS groups only; Mann–Whitney U-test (continuous variables), *χ*^2^-test (categorical variables). ^a^ Includes patients who survived. ^b^ Infection type: chest 86%; blood 14%; urinary tract; 19%; wound 24%. BD—base deficit; CSL—crystalloid (mL); FD—free days; GCS—Glasgow Coma Score; ISS—Injury Severity Score; HR—heart rate; MHP—major hemorrhage protocol; MODS—multiple organ dysfunction syndrome; RBC—red blood cells; SBP—systolic blood pressure; TBI—traumatic brain injury; TTR—time to recovery; and TXA—tranexamic acid.

**Table 2:** Whole blood lymphocyte subset counts. Values are median with interquartile range.

	Cell Counts (×10^8^/L)					*p* Value	
	HV	≤2 h	24 h	72 h	HV v. ≤2 h	≤2 h v. 24 h	≤2 h v. 72 h
**Lymphocytes**	18 (16–23)	21 (17–32)	12 (9.0–16)	9 (6.5–12)	0.196	**<0.001**	**<0.001**
**NK cells**	2.1 (1.2–2.9)	4.6 (2.7–7.1)	0.76 (0.42–1.4)	0.58 (0.35–0.95)	**<0.001**	**<0.001**	**<0.001**
**NK dim**	2.0 (1.2–3.2)	3.5 (2.2–5.6)	0.69 (0.32–1.2)	0.55 (0.4–1.0)	0.004	**<0.001**	**<0.001**
**NK bright**	0.14 (0.11–0.17)	0.20 (0.14–0.3)	0.08 (0.05–0.10)	0.05 (0.04–0.08)	0.068	**<0.001**	**<0.001**
**V** **δ** **1 cells**	0.09 (0.05–0.17)	0.12 (0.04–0.43)	0.04 (0.02–0.08)	0.04 (0.01–0.07)	0.348	**<0.001**	**<0.001**
**V** **δ** **2 cells**	0.46 (0.21–0.58)	0.24 (0.10–0.51)	0.14 (0.07–0.16)	0.09 (0.05–0.15)	0.074	**0.003**	**<0.001**
***i*** **NKT cells**	0.01 (0.00–0.03)	0.01 (0.00–0.01)	0.00 (0.00–0.01)	0.00 (0.00–0.00)	0.062	**0.024**	**0.002**
**B cells**	1.3 (0.68–2.4)	0.87 (0.61–1.5)	1.2 (0.51–2.0)	0.62 (0.51–1.2)	0.252	0.487	0.159
**CD4 cells**	11.0 (8.3–12.5)	6.7 (4.5–9.7)	6.1(3.2–8.9)	3.9 (2.9–5.7)	**0.002**	0.387	**0.022**
**CD8 cells**	3.9 (3.3–5.4)	4.5(2.3–6.7)	2.1 (1.3–3.4)	1.2 (0.81–2.0)	0.854	0.051	**<0.001**

HV vs. ≤2 h, Mann–Whitney U-test; ≤2 h vs. 24 h, and ≤2 h vs. 72 h, Wilcoxon signed rank test. HV—healthy volunteer; NK—natural killer; Vδ—gamma delta.

## Data Availability

The original contributions presented in this study are included in the article/Supplementary Materials. Further inquiries can be directed to the corresponding author.

## Supplementary Materials

The following supporting information can be downloaded at: https://www.mdpi.com/article/10.3390/biom16091228/s1, Figure S1: Gating strategy for innate and adaptive lymphocytes; Figure S2: Trauma patient flow cytometry cohort recruitment flow diagram; Figure S3: Representative flow cytometry plots showing changes in lymphocyte subsets over time in patients with and without MODS, and early death; Figure S4: Relationship between NK subsets and receptors and adverse outcome; Table S1: Flow cytometry panels used in lymphocyte immunophenotyping studies; Table S2: Antibodies uses for flow cytometry analysis; Table S3: Demographics and outcomes for the five clusters (C1–C5); Table S4: Characteristics of the proteomics cohort.

## Abbreviations

The following abbreviations are used in this manuscript:

BD	Base deficit
CD	Cluster of differentiation
CSL	Crystalloid
FD	Free days
GCS	Glasgow Coma Score
HR	Heart rate
HV	Healthy volunteer
*i*NKT	Invariant natural killer T cells
IQR	Interquartile range
ISS	Injury Severity Score
LOS	Length of stay
MHP	Major hemorrhage protocol
MODS	Multiple organ dysfunction syndrome
NK	Natural killer
PRMODS	Prolonged multiple organ dysfunction syndrome
RBC	Red blood cells
SBP	Systolic blood pressure
TBI	Traumatic brain injury
TTR	Time to recovery
TXA	Tranexamic acid
Vδ	Gamma delta
WCC	White cell count
