# Supplementary material for: Natural Killer Cells Dominate the Hyperacute Lymphocyte Response to Major Trauma and Are Associated with Organ Dysfunction
Source: Biomolecules. 2026 Aug 24;16(9):1228. doi: 10.3390/biom16091228 (PMC13604584; doi:10.3390/biom16091228)
Supplement: Supplementary file 1 [file biomolecules-16-01228-s001.zip › biomolecules-4425036 supplementary.pdf]

SUPPLEMENTAL MATERIAL

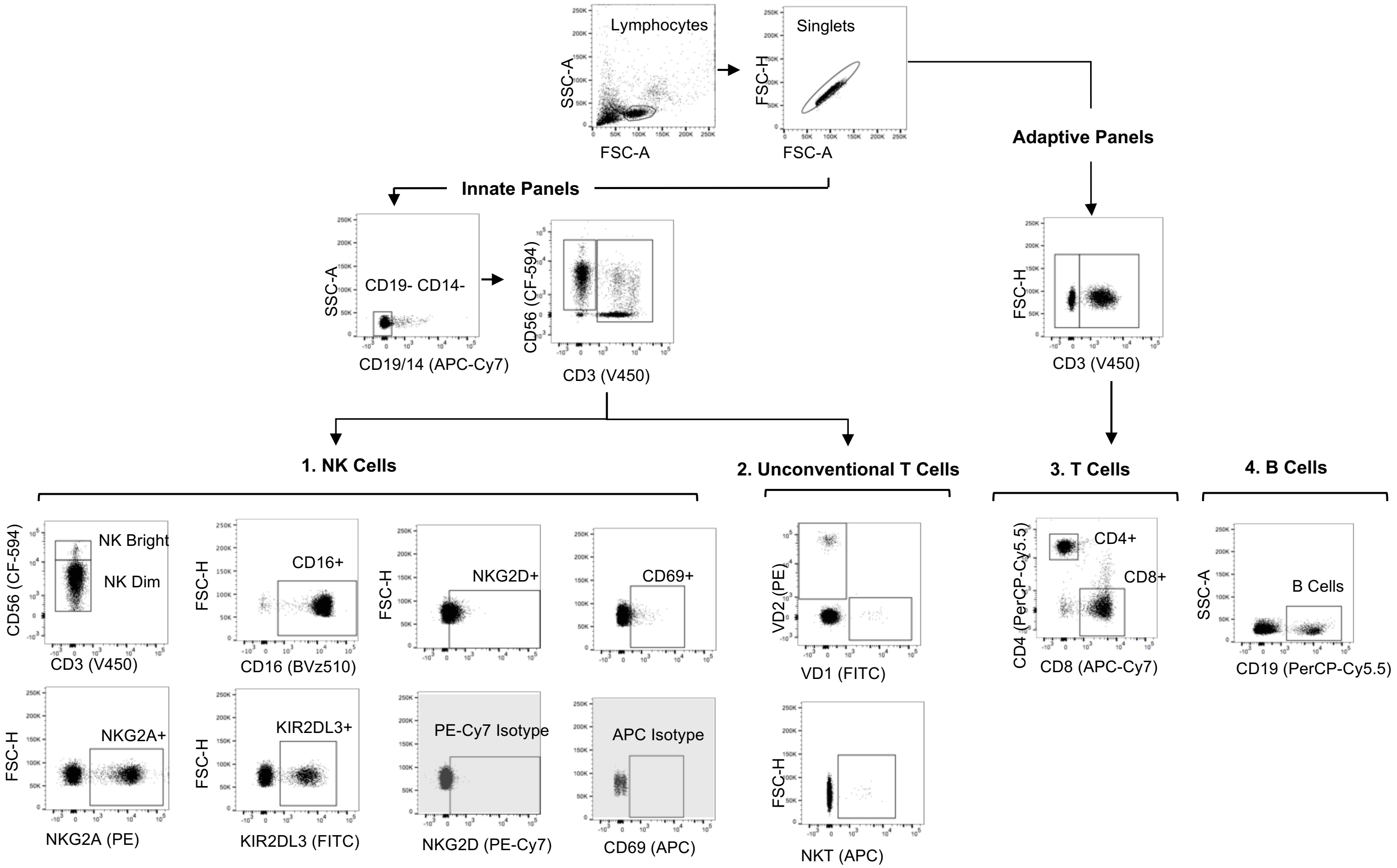

Supplementary Figure S1: Gating strategy for innate and adaptive lymphocytes. Lymphocytes were gated on forward and side scatter, and doublets were excluded. For the innate panel, B cells and monocytes were subsequently removed using CD19/14. NK cells were gated as CD56+CD3-, and from this population, KIR2DL3+, NKG2A+, NKG2D+, CD69+, CD16+ cells were identified against forward scatter. Isotype controls were used to assist gate placement for NKG2D and CD69. V**δ1**, Vδ2, and iNKT gates were applied to the CD3+ population. Figures in grey represent controls. For the adaptive panel CD4+ and CD8+ T cells were initially identified within the CD3+ lymphocyte population. B cells were identified from the CD3- population using CD19.

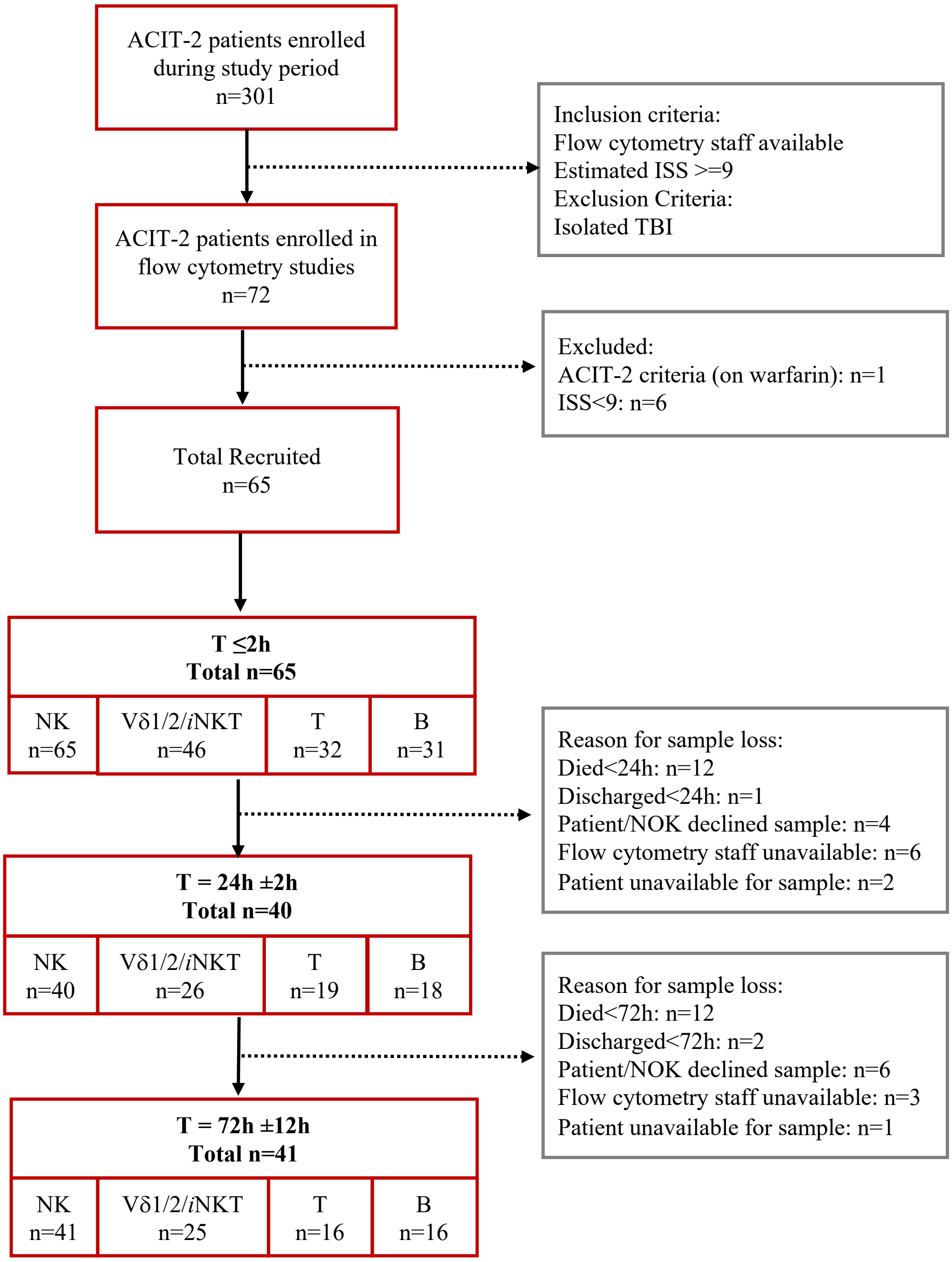

Supplementary Figure S2. Trauma patient flow cytometry cohort recruitment flow diagram. Flow diagram illustrating patients recruited for natural killer (NK), gamma delta type 1 and 2 (Vδ1 and Vδ2), natural killer T (iNKT), B and T cell flow cytometry studies at ≤2 h, 24 h and 72 h after injury (red boxes). Reasons for participant exclusion or sample loss are illustrated in grey boxes. NOK – next of kin.

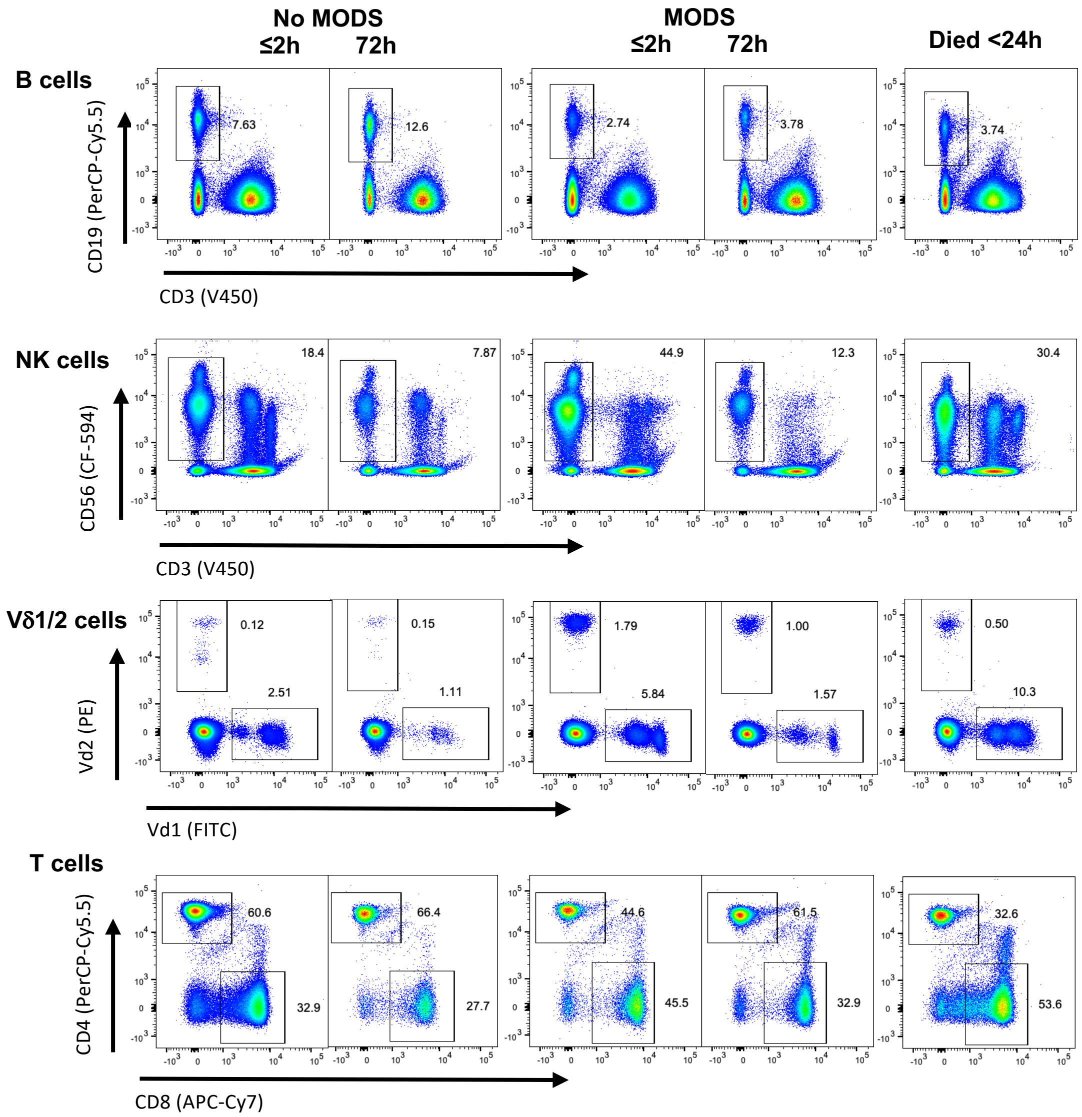

Supplementary Figure S3. Representative flow cytometry plots showing changes in lymphocyte subsets over time in patients with and without MODS, and early death. Representative flow cytometry plots showing proportionate changes in NK cells, Vδ1/Vδ2 cells, CD4+ T/CD8+ T cells, and B cells in a patient with No MODS (ISS 18, BD 5.7), MODS (ISS 54, BD 10) and who died<24 h (ISS 42, BD29). BD – base deficit; HV – healthy volunteer; ISS – injury severity score; MODS – multiple organ dysfunction syndrome; NK – natural killer; Vδ - gamma delta

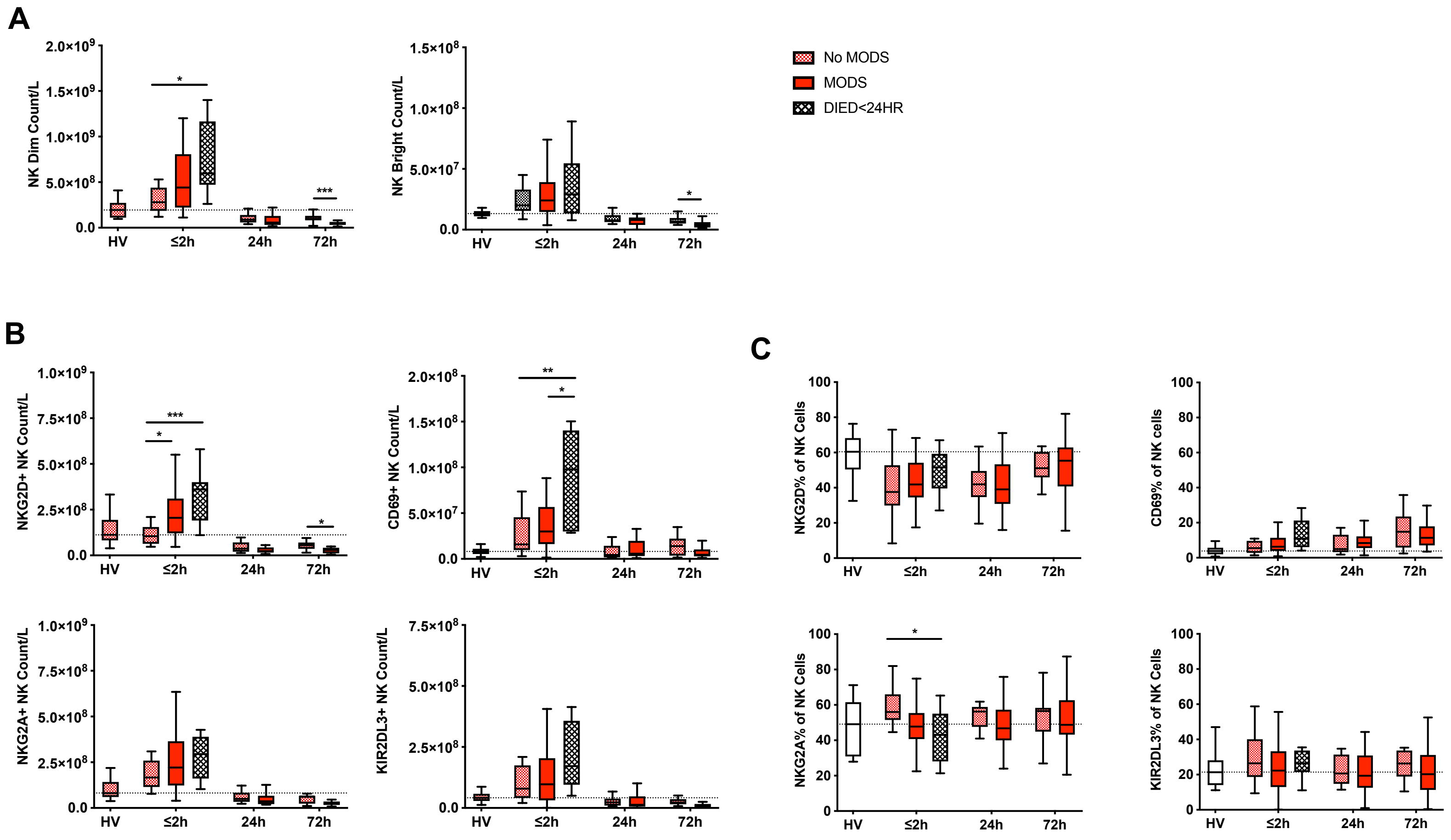

Supplementary Figure S4. Relationship between NK subsets and receptors and adverse outcome. (A) Absolute counts of NK Dim and NK Bright subsets. (B) Absolute counts and (C) Percentages of NK activation (NKG2D & CD69) and inhibition receptors (NKG2A & KIR2DL3). *p<0.05, **p<0.01, ***p<0.001; ≤2 h Kruskal-Wallis test with Dunn's post hoc corrections comparing No MODS vs. MODS vs. early deaths; 24 h & 72 h Mann Whitney U-test comparing No MODS vs. MODS. HV – healthy volunteer; MODS – multiple organ dysfunction syndrome; NK – natural killer.

SUPPLEMENTAL TABLES

Supplementary Table S1: Flow cytometry panels used in lymphocyte immunophenotyping studies.

Fluorochrome	Receptor (Clone)			
	NK	Unconventional T	T	B
**FITC**	KIR2DL3/CD158b (REA147)[a]	Vd1 (TS8.2)[b]	-	-
**PE**	NKG2A/CD159a (REA110)[a]	Vd2 (B6)[c]	CCR7 (150503)*	CD24 (ML5)
**PE-CF 594**	CD56 (NCAM 16.2)	CD56 (NCAM 16.2)	-	-
**PerCP**	-	-	-	-
**PerCP-Cy5.5**	-	-	CD4 (SK3)	CD19 (HIB19)
**PE-Cy7**	NKG2D/CD314 (1D11)*	-	CD45RA (L78)	CD27 (M-T271)*
**APC**	CD69 (L78)*	Va24 (6B11)[c]	CD38 (HB7)*	CD38 (HB7)*
**APC-H7**	-	-	CD8 (SK1)	CD20 (2H7)
**APC-Cy7**	CD14 (MoP3) CD19 (SJ25CI)	CD14 (MoP3) CD19 (SJ25CI)	-	-
**V450**	CD3 (UCHT1)	CD3 (UCHT1)	CD3 (UCHT1)	CD3 (UCHT1)
**V500**	-	-	HLA-DR (G46-6)*	-
**BV510**	CD16 (3G8)	CD16 (3G8)	-	IgD (IA6-2)*
**BV605**	-	-	-	-
**BUV395**	-	-	-	-

APC – allophycocyanin; BV – Brilliant Violet; BUV – Brilliant Ultra-Violet; CD – cluster of differentiation; CR – chemokine receptor; Cy7 – cyanine 7 tandem; FITC – fluorescein isothiocyanate; PE – phycoerythrin; PerCP – peridinin chlorophyll protein. *Control tube using isotype or fluorescence-minus-one to assist with gating of positive and negative population. All fluorochromes were purchased from BD except [a]Miltenyi Biotech, [b]Abcam, [c]Biolegend.

Supplementary Table S2: Antibodies uses for flow cytometry analysis

Antigen	Fluorochrome	Clone	Company	Catalogue Number
CD56	CF-594	NCAM 16.2	BD	564849
CD3	V450	UCHT1	BD	560365
CD16	BV510	3G8	BD	563830
CD14	APC-Cy7	MoP3	BD	557831
CD19	APC-Cy7	SJ25CI	BD	557791
CD69	APC	L78	BD	340560
CD69 Isotype	APC	MOPC-21 (Mouse IgG1, k)	BD	555751
NKG2D	PE-Cy7	1D11	BD	562365
NKG2D Isotype	PE-Cy7	Ms IgG1, κ Iso Ctrl	BD	557646
Vδ1	FITC	TS8.2	Abcam	ab171097
Vδ2	PE	B6	Biolegend	331408
Vα24	APC	6B11	Biolegend	342908
NKG2A/CD159a	PE	REA110	Miltenyi Biotech	130–098–814
KIR2DL3/CD158b	FITC	REA147	Miltenyi Biotech	130–100–125
CD4	PerCP-Cy5.5	SK3	BD	566924
CD8	APC-H7	SK1	BD	560197
CD19	PerCP-Cy5.5	HIB19	BD	561295

APC – allophycocyanin; BD – Becton Dickinson; BV – Brilliant Violet; Cy7 – cyanine 7; FITC – fluorescein isothiocyanate; HIB19 – human B-cell clone; KIR2DL3 – killer cell immunoglobulin-like receptor 2DL3; NKG2D – natural killer group 2D; PE – phycoerythrin; PerCP – peridinin chlorophyll protein

Supplementary Table S3: Demographics and outcomes for the five clusters (C1-C5).

	C1	C2	C3	C4	C5
**N**[†]	5	8	14	13	12
**Demographics**					
Age	46 (44-62)	33 (23-54)	36 (28-48)	46 (24-54)	37 (31-53)
Male	60% (3)	75% (6)	86% (12)	77% (10)	83% (10)
**Injury Characteristics**					
Blunt Mechanism	100% (5)	63% (5)	79% (11)	77% (10)	92% (11)
ISS	17 (17-29)	17 (15-25)	34 (16-41)	29 (9-34)	34 (21-44)[h]
TBI	25% (1/4)	0%	29% (4)	23% (3)	25% (3)
**Baseline Values**					
HR (beats/min)	99 (81-115)	87 (74-112)	114 (90-125)	110 (100-126)	102 (78-135)
SBP (mmHg)	120 (60-124)	124 (112-133)	96 (70-125)	126 (124-146)[g]	92 (65-117)[j]
GCS	8 (8-8)	15 (15-15)	12 (7-14)[e]	14 (14-15)	10 (3-14)[h, j]
pH	7.3 (7.2-7.3)	7.4 (7.3-7.4)	7.2 (7.0-7.3)[e]	7.3 (7.2-7.3)	7.2 (7.0-7.3)[h, j]
BD	7.3 (4.5-9.2)	2.0 (0.3-5.1)	9.4 (1.9-19.3)	3.7 (2.3-7.2)	13 (6.0-17.0)[h, j]
**Interventions**					
MHP	100% (5)	50% (4)	79% (11)	85% (11)	92% (11)[h]
TXA	80% (4)	50% (4)	79% (11)	85% (11)	92% (11)[h]
Pre-baseline RBC	1 (0-3)	0 (0-2)	1 (0-3)	1 (0-2)	3 (1-5)
Pre-baseline CSL	500 (0-750)	0 (0-0)	375 (0-800)[e]	150 (0-400)	0 (0-175)
24 h RBC	2 (1-3)	2 (0-7)	9 (2-18)	2 (0-5)	6 (5-11)[h]
24 h CSL	4250 (2250-5350)	2525 (885-3265)	3688 (2650-4350)	2234 (600-3837)	1416 (400-3939)
**Outcomes**					
Mortality (n)	20% (1)	0%	29% (4)	8% (1)	33% (4)
TTR	6 (4-16)	0 (0-1)	12 (1-30)[e]	4 (0-6)	12 (4-30)[h]
MODS (n)	75% (3/4)	25% (2/8)	83% (10/12)[e]	58% (7/12)	75% (6/8)[h]
PRMODS (n)	25% (1/4)	0%	42% (5/12) [e]	8% (1/12)	25% (2/8)
Infection (n) [‡]	50% (2/2)	13% (1/8)	50% (6/12)	33% (4/12)	63% (5/8)[h]
Ventilator FD	24 (17-26)	30 (29-30)	23 (0-30)	28 (24-30)	22 (0-27)[h, j]
Critical Care FD	16 (6-23)	28 (26-30)	12 (9-28)[e]	25 (18-28)	12 (0-22)[h, j]
Hospital FD	4 (0-8)	13 (2-23)	0 (0-21)	6 (0-21)	0 (0-13)

Data presented as median (interquartile range) unless specified. Mann-Whitney U-test (continuous variables), χ2-test (categorical variables). Statistical significance $p<0.05$ for: [a]C1 vs. C2; [b]C1 vs. C3; [c]C1 vs. C4; [d]C1 vs. C5; [e]C2 vs. C3; [f]C2 vs C4; [g]C3 vs. C4; C2 vs. C5[h]; [i]C3 vs. C5; [j]C4 vs. C5.

[†]Excludes the 2 patients that did not cluster into C1-5 [‡]Includes patients who survived.

Supplementary Table S4: Characteristics of the proteomics cohort.

	All	No MODS	MODS	Died<24 h	p-value[1]
**N**	412	317	83	12	
**Demographics**					
Age	37 (28-50)	37 (28-50)	44 (32-66)	56 (37-66)	**0.008**
Male, n (%)	333 (81)	251 (79)	74 (89)	8 (67)	0.27
**Injury Characteristics**					
Blunt mechanism, n (%)	412 (100)	317 (100)	83 (100)	12 (100)	>0.99
ISS	11 (4-25)	9 (4-19)	29 (17-35)	29 (25-47)	**<0.001**
TBI, n (%)	116 (28)	54 (17)	56 (63)	6 (50)	**<0.001**
**Baseline Values**					
HR (beats/min)	85 (71-102)	85 (71-102)	98 (77-120)	115 (92-145)	**<0.001**
SBP (mmHg)	135 (121-151)	135 (121-151)	121 (98-150)	104 (63-156)	**0.002**
GCS	15 (13-15)	15 (13-15)	9 (5-14)	4 (3-13)	**<0.001**
Lactate (mmol/L)	1.8 (1.2-2.6)	1.8 (1.2-2.6)	2.6 (1.5-4.4)	6.1 (2.1-8.3)	**<0.001**
BD (mmol/L)	0.1 (-1.5-1.9)	0.1 (-1.5-1.9)	3.1 (1.3-5.8)	8.7 (6.3-13.3)	**<0.001**
**Interventions**					
MHP, n (%)	46 (11)	12 (4)	27 (33)	7 (58)	**<0.001**
24 h RBC	0 (0-0)	0 (0-0)	0 (0-4)	8 (1-19)	**<0.001**
24 h CSL	1025 (0-2575)	679 (0-2000)	3130 (2000-4497)	1425 (350-4500)	**<0.001**
**Outcomes**					
28 d Mortality, n (%)	23 (6)	2 (1)	9 (11)	12 (100)	**<0.001**
24 h Mortality, n (%)	12 (3)	0 (0)	0 (0)	12 (100)	-
Ventilator FD	28 (27-28)	28 (28-28)	21 (9-26)	-	**<0.001**
Critical care FD	28 (26-28)	28 (28-28)	16 (4-23)	-	**<0.001**

Data presented as median (interquartile range) or n (%).

[1]Kruskal-Wallis test (continuous variables) or Mann-Whitney U-test (ventilator FD and Critical care FD), Fisher's exact test (categorical variables).
